# Network-driven cancer cell avatars for combination discovery and biomarker identification for DNA damage response inhibitors

**DOI:** 10.1038/s41540-024-00394-w

**Published:** 2024-06-21

**Authors:** Orsolya Papp, Viktória Jordán, Szabolcs Hetey, Róbert Balázs, Valér Kaszás, Árpád Bartha, Nóra N. Ordasi, Sebestyén Kamp, Bálint Farkas, Jerome Mettetal, Jonathan R. Dry, Duncan Young, Ben Sidders, Krishna C. Bulusu, Daniel V. Veres

**Affiliations:** 1 Turbine Simulated Cell Technologies, Budapest, Hungary; 2 Oncology Bioscience, Research and Early Development, Oncology R&D, AstraZeneca, Waltham, MA USA; 3 Early Data Science, Oncology Data Science, Oncology R&D, AstraZeneca, Waltham, MA USA; 4 Search and Evaluation, Oncology R&D, AstraZeneca, Cambridge, UK; 5 Early Data Science, Oncology Data Science, Oncology R&D, AstraZeneca, Cambridge, UK

**Keywords:** Computational biology and bioinformatics, Computer modelling, Cancer

## Abstract

Combination therapy is well established as a key intervention strategy for cancer treatment, with the potential to overcome monotherapy resistance and deliver a more durable efficacy. However, given the scale of unexplored potential target space and the resulting combinatorial explosion, identifying efficacious drug combinations is a critical unmet need that is still evolving. In this paper, we demonstrate a network biology-driven, simulation-based solution, the Simulated Cell™. Integration of omics data with a curated signaling network enables the accurate and interpretable prediction of 66,348 combination-cell line pairs obtained from a large-scale combinatorial drug sensitivity screen of 684 combinations across 97 cancer cell lines (BAC = 0.62, AUC = 0.7). We highlight drug combination pairs that interact with DNA Damage Response pathways and are predicted to be synergistic, and deep network insight to identify biomarkers driving combination synergy. We demonstrate that the cancer cell ‘avatars’ capture the biological complexity of their in vitro counterparts, enabling the identification of pathway-level mechanisms of combination benefit to guide clinical translatability.

## Introduction

Genetic and phenotypic heterogeneity within a nest of primary tumor cells and their microenvironment has long been one of the major challenges in designing successful and curative treatment plans for cancer patients. The phenomenon contributes greatly to the rapid development of drug resistance and subsequent disease progression^1–3^. Selecting the appropriate therapy option by optimizing for personalized biomarker patterns increases the likelihood of efficacy^4–6^, but most cancers progress and subsequential therapy options are needed to tackle disease progression.

Most aggressive human malignancies share common characteristics like aberrant cell proliferation resulting in high replication stress and functional defects in DNA-damage repair (DDR) pathways^7,8^. The loss of well-functioning DDR has a crucial involvement in uncontrolled tumor growth, disease progression, and therapeutic responses^9^. Several studies have proved the therapeutic opportunity of targeting DDR in highly aggressive cancers in the clinic^10–12^, however, patients show variable and often short-lived benefits.

Drug combinations hold the promise of providing a more durable efficacy on cancer cells while managing additive toxicity by decreasing the dosages of individual therapies^13^. However, finding the right combination partners in the right therapeutic window with potential translatability to the clinic is challenging, therefore, novel approaches are needed to identify combinations that could be ultimately beneficial for patients^14,15^. One such example includes linking the synergistic manner of a combination with the molecular features of the given tumor mass^16,17^. In clinical trials, the molecular mechanism determining synergy is often unrecognized, and combinations are often only effective in a patient subset^18^.

Computational biology is fundamental in developing biological knowledge that translates into clinical practice by revealing potential biomarkers that can predict clinical outcomes and narrowing down the enormous space of potentially synergistic drug combinations^8,19,20^. Understanding the mechanism of how drug interventions can modify each other could change the perception and prediction of beneficial therapies. Currently, available drug combination prediction methods range from mathematical predictions based on chemical features to pathway-level assessments of biological relations^21,22^ but display their own limitations. These limitations include: (i) lack of explanatory mechanism, (ii) uncomprehensive capture of biological feature space, and (iii) uncaptured treatment effect at a pathway level. By integrating complementary data reflecting different levels of biological understanding, it is possible to generate a more scalable system of drug therapy prediction with a more granular view of the underlying mechanism.

Network-based approaches are emerging as powerful tools for studying the complexity of cancerous diseases by observing functional interactions between molecules through an interaction network to aid molecular interpretation of the cellular mechanisms behind synergy. These networks have the power to reveal relationships between individual nodes in a certain molecular pathway but also can decipher a higher-level assembly of pathway cross-talk and interconnected cellular mechanisms^23,24^. To understand the intricate dynamics of cell signaling networks, Kholodenko et al. presented one of the pioneering dynamical models of cell signaling^25^; a topology-dependent systematic analysis of MAPK signaling reactivation and its potential to initiate resistance mechanisms against RAF inhibitors. In recent papers, the authors used a similar way to mathematically model and understand how allosteric inhibitors affect kinase dimerization and develop an advanced, rule-based dynamic model to assess various combinations of structurally diverse RAF inhibitors, aiming to effectively inhibit MEK/ERK signaling^26^. This model was able to forecast that RAF inhibitor combinations are capable of inhibiting ERK activity in the presence of oncogenic RAS and/or BRAFV600E backgrounds^27^. These models, albeit small-scale and specialized, serve to elucidate biological phenomena. However, without predictive capabilities, this restricts their application in conducting large-scale inhibitor screening sweeps and reduces the feasibility of industrial applications. While retaining the advantages of mechanistic interpretation is desirable, these models need to scale to emulate in vitro experiments and enhance predictive capabilities. There are other initiatives engaged in larger scale modeling of drug responses in cancer cell lines and can serve as pertinent precursors to our methodology. One of these could predict the effect of drug combinations from single drug data and benchmarked their results also against top performers of the DREAM challenge. However, the model is still relatively small in terms of the size of the used train set, consisting of 120 cell lines and 7 drugs^28^. Similar challenges apply to the Erdem et al. model, also primarily utilized for predicting drug combinations^29^. Their model employs an ordinary differential equation (ODE) framework and described as large-scale; however, it encounters computational cost issues beside the fact their deterministic module encompasses the dynamics of only 774 proteins, protein complexes, and post-translationally modified species ending up in 2449 reactions.

**Fig. 1:**
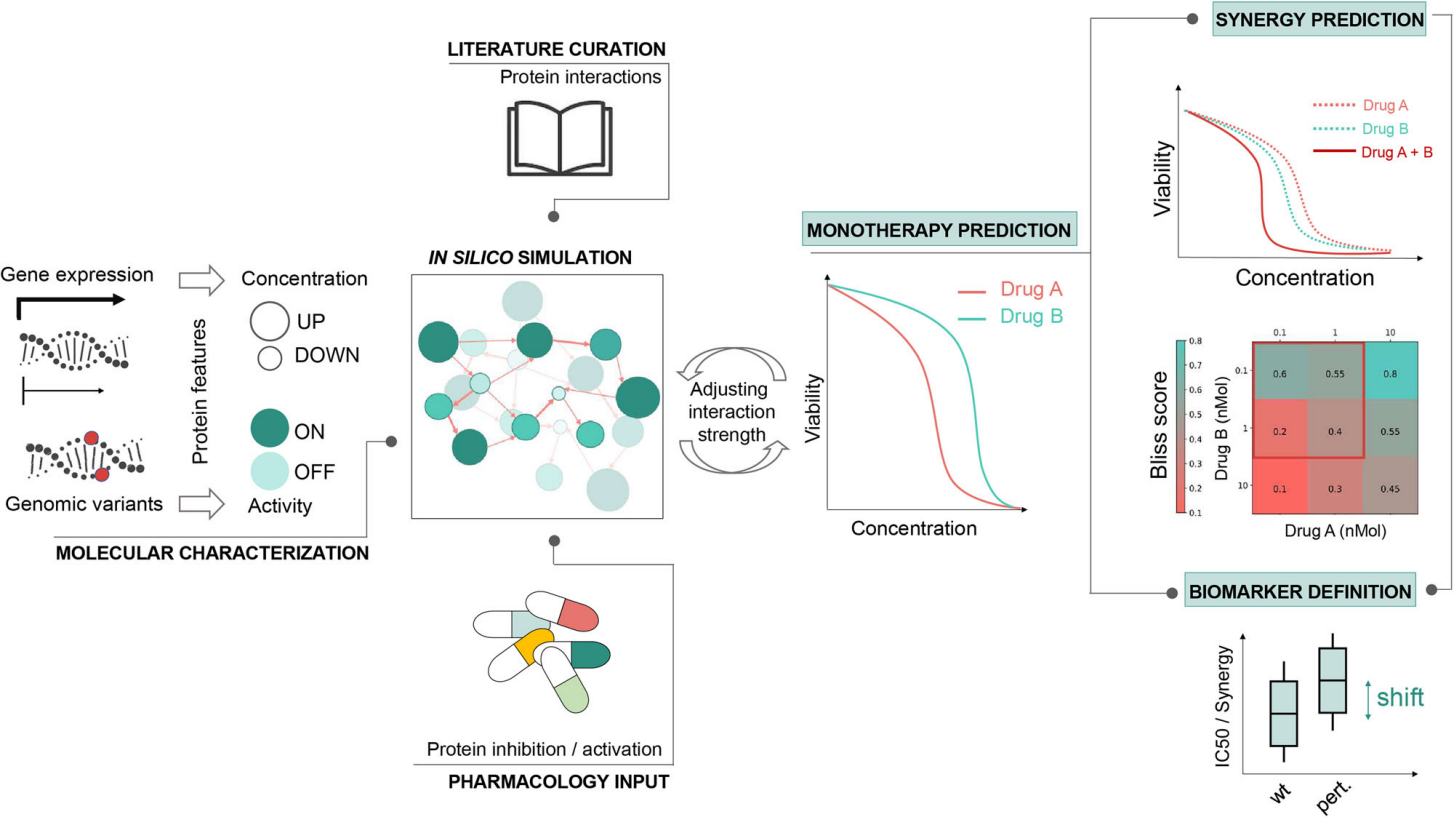
Integration of diverse data types to feed our Simulated Cell to elucidate drug-specific biomarkers as well as to help understand the mechanism of predicted synergy. By using cell line-specific genomic and transcriptomic data, the Simulated Cell can be transformed from a general wiring diagram to a network-based in silico replica of a cancer cell line. It carries its characteristic mutations beside cell line-specific expression patterns which modify the effect of a node by the binary activity and continuous concentration parameter changes, respectively. Available molecular compound target profiles enable pharmacological perturbation of the signaling network in a dose-dependent and compound-specific manner by modifying the concentration parameter of primary and off-targets of drugs included in the protein-protein interaction network. This approach generates insights into the activity of affected pathways after drug exposure and predicts the cell line’s response to a given intervention by calculating IC50 values and dose-dependent changes in viability. Each Simulated Cell is calibrated to match its in vitro counterpart’s IC50 value accurately. This is achieved by setting up the adequate and proportionate contribution of each regulator of a given protein so biological hypotheses are recapitulated on the level of protein interactions that leads to the accurate pathway level signal propagation, and ultimately correct survival response. After the calibration process to establish the network parameters and enable in silico experiments, cell line-specific responses of the Simulated Cell to a certain combination therapy–measured by combination synergy and combination-specific viability–are strictly trained on the monotherapeutic effect of the combination partners. By inducing artificial protein alterations, affecting protein activity and/or concentration, combination-specific biomarkers and their effect on combination synergy and cell viability can be identified.

In this study, we used a solution called the Simulated Cell from Turbine Ltd^30^. which integrates the interpretability inherent in mechanistic signaling network models with the necessary scalability to conduct simulations capable of facilitating large-scale drug screens and providing in silico insights. Simulated Cell simulates the signal propagation in a signaling network customized for cancer cells to generate interpretable synergy predictions for combinations of DDR-targeting drugs with other agents. By using a primarily protein-protein interaction-based signaling network and cell-type-specific multi-omics gene-level data features that are thereafter simulated using difference equations under discrete time, we were able to examine treatment effects at a pathway level (“Methods”, Fig. 1). First, we use the in vitro monotherapy measurements as benchmarks for the in silico cell during manual calibration that aims to match their phenotypes and bring their monotherapy measurements and predictions close to each other. This method enables de novo combination synergy prediction. We calculated Bliss independence model-based synergy scores for ~66,000 cell line-combination pairs. The in silico results were analyzed through a transparent benchmarking procedure using experimental endpoints of in vitro measurements published in the DREAM Drug Combinations Challenge^17^.

The Simulated Cell enabled us to interpret the biological rationale determining predictions by following intracellular signal propagation from molecule to molecule, originating from the intervened drug targets to the effector nodes determining cell fate decisions. Furthermore, nodes in the Simulated Cell network could be systematically perturbed in a dose-dependent manner. These perturbations helped the identification of important, non-trivial regulators of the combination-specific response. We used these tools to identify combination-specific biomarkers for poly (ADP-ribose) polymerase (PARP) inhibition combined with ataxia telangiectasia mutated kinase (ATM) inhibition and DNA-dependent protein kinase subunit c (PRKDC) inhibition combined with nuclear factor-kappa B (NFKB) repression while also demonstrating their significant effect on the level of synergy and cell death.

Our findings demonstrate the value of the Simulated Cell (model version 4 used in this study) as an interpretable network biology-based framework for predicting synergistic drug combination mechanisms. The predictions derived utilizing this toolset could help deliver more precise hypotheses for prospective validation experiments and accelerate the critical translation of in silico/in vitro findings to the clinic.

## Results

### Simulated Cell effectively models mechanism of actions for DDR-targeting and some non-DDR-targeting drugs

**Fig. 2:**
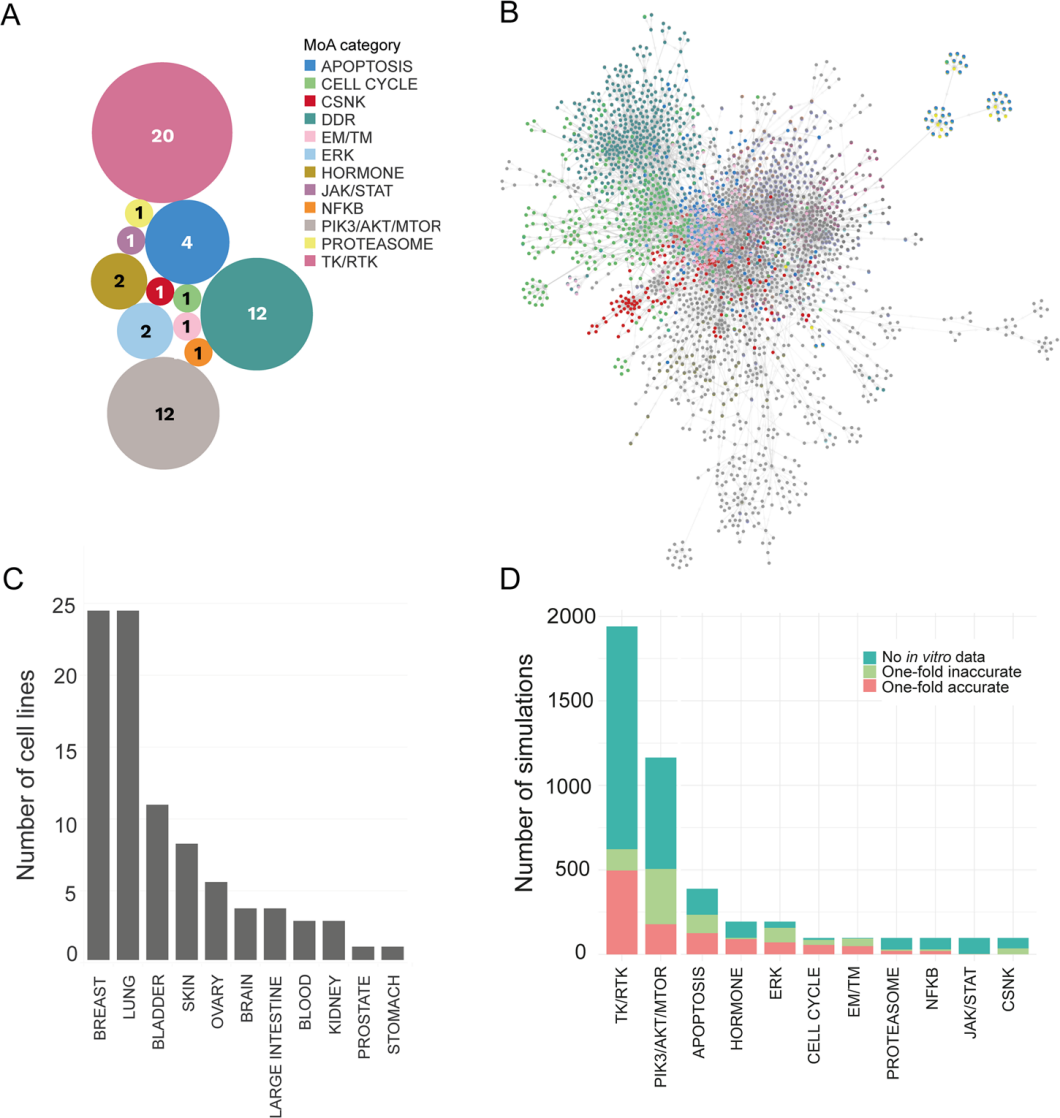
The simulated cell encyclopedia and proportions of accurate and inaccurate simulations across the predicted monotherapy landscape. **A** Compound library of the presented combinations. The compounds were categorized into MoA groups based on the pathway membership of primary drug targets with the lowest binding affinity. **B** Snapshot of the network functioning in the Simulated Cell. Our Simulated Cell is based on a graph, where proteins and cellular events are represented as nodes and the physicochemical properties of their interactions are represented as edges between them. DDR pathway members are highlighted in coral, while survival-related pathways, such as different parts of the cell cycle, are colored turquoise. In the model version used for this analysis, our network included 56 modules covering the main cancer-driving pathways with a total of 1997 nodes and 5004 interactions, out of which 14 modules cover the DDR-related mechanisms. **C** Indications covered by cell lines during in silico experiments. The cell lines were grouped into indications according to the localization of the primary tumor which the cell line was derived from. **D** Proportion of accurate and inaccurate in silico monotherapy predictions compared to in vitro measurements (from public sources, not from the DREAM dataset) in mechanism of action (MoA) categories. The Y-axis shows the overall number of simulations, regardless of the in vitro value availability and mechanism of action groups are ranked by the number of one-fold accurate predictions. Unfortunately, due to the low in vitro data coverage, there are many cases where benchmarking was not possible. Accurate range was defined as |log10(IC50_in vitro_) - log10(IC50_in silico_)| <1), meaning the measured in vitro IC50 is in the range of (IC50_in silico_ / 10, *IC50*_in silico_ * 10) covering an interval of two orders of magnitude. This level of accuracy reflects the variability between two in vitro data points in repeated experiments. On the MoA level, the most accurate groups were the TK/RTK and PIK3-AKT-MTOR. CSNK Casein kinase, DDR DNA damage repair, EM/TM Epigenetic/Transcriptomic modulation, ERK extracellular signal-regulated kinase, JAK/STAT Janus kinase/signal transducers and activators of transcription, NFKB NF-kappa B, PIK3/AKT/MTOR Phosphatidylinositol 3’ -kinase(PI3K)-AKT-mTOR, TK/RTK tyrosine kinase/receptor tyrosine kinase.

Monotherapy in silico experiments were performed on 97 cell lines covering 11 indications (Fig. 2C) with a total of 12 DDR and 46 non-DDR targeting drugs (Fig. 2A). We supplemented the in vitro measurements of the DREAM Challenge^17^ with further data points from various cellular response databases (Methods; Supplementary Data 1; Supplementary Methods 1) as objective experimental endpoints. Comparing the DREAM data to other sources, we observed overall increased sensitivity, probably due to differences in the length of treatment time (DREAM 120 h vs. Miscellaneous 72 h) (Supplementary Fig. 3).

The drug response of each simulated cell line model was calibrated to reflect their in vitro counterparts (Methods; Supplementary Data 1). During the calibration of the Simulated Cell for the more valuable mechanistic explanations, the priority is to create a correct signaling cascade on a granular, protein level, but only within the limits of keeping an admissible IC50 fit on the majority of the drugs. Due to the size of the network, and within the number of not modeled proteins from the proteome, not all drugs can be fitted in silico according to their in vitro IC50 values (Fig. 2D). To test the accuracy of this in silico model, receiver operating characteristic (ROC) analysis was applied to the monotherapy response results (Methods). When the results of the individual DDR and non-DDR targeting therapies were aggregated, overall AUC values were 0.7 and 0.47 respectively, with varying performance between individual drugs, especially in the non-DDR category (Supplementary Fig. 4).

### DDR:DDR drug combinations displayed higher synergy than DDR:non-DDR targeting pairs

An in silico combinatorial drug sensitivity screen of 684 combinations (12 DDR drugs combined with each other and 46 non-DDR drugs without combining the drugs with themselves) (Fig. 2A, Supplementary Data 1) was conducted, each simulated in a 16-by-16 dose matrix within a 0–10,000 nmol dose range across 97 cancer cell lines, resulting in 66,348 combination-cell line pairs measured in total. The generated dataset included in silico dose-dependent cell viability predictions and Bliss independence synergy scores^31^ for all combinations (Methods, Supplementary Methods 2; Supplementary Data 2).

Each cell line-specific combination grid underwent a quality control process to filter out erroneous predictions that do not conform to dose-response principles for one or both compounds (Supplementary Methods 1). Out of the 66,348 cell line-specific combinations, 66,200 combinations remained after quality control and were included in further analytical steps, and only 116 combination grids were discarded due to low quality (Supplementary Fig. 5). After the discretization of Bliss scores, we were able to distinguish between strongly, moderately, and non-synergistic pairwise combinations (Table 1, Benchmarking of the predicted combination synergy scores to experimental data; Supplementary Fig. 6).

**Table 1:** Thresholds of synergy categories determined by K-means clustering

	Non-synergistic	Moderately synergistic	Strongly synergistic
Interval of in silico synergy scores	Bliss score <0.24	0.24 < Bliss score <0.67	Bliss score >0.67
Number of combination-cell line pairs	47583	5212	13437
Percentage of combination-cell line pairs	71.84%	7.86%	20.8%

Most of the combination-cell line pairs were not synergistic based on the low in silico Bliss values, while the majority of the remaining combinations (20.8% of all) were considered to be strongly synergistic.

**Fig. 3:**
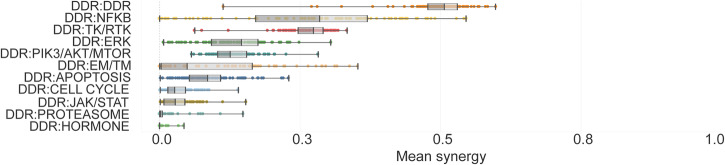
Synergy distribution in different combination MoA group categories. Combination-cell line pairs were categorized into MoA groups based on the molecular targets of the compounds with the lowest binding affinity value. Combination MoA groups were sorted based on the calculated mean synergy. We observed a generally higher synergy in the case of DDRi:DDRi combinations, compared to pairs where DDR inhibition was combined with compounds acting beyond DDR.

The distribution of synergistic and non-synergistic combinations was similar across tumor types. Regarding drug mechanism of action groups, DDR:DDR targeting drug combinations had 2.95-fold higher mean synergy than DDR:non-DDR targeting combinations (0.4944 vs. 0.1672, respectively) (Fig. 3). DDRi:DDRi intrapathway synergy is a logical and known phenomenon. As an example, in BRCA2 mutant high-grade serous ovarian cancer PDX cells, the combinations of PARPi with ATRi or CHKi were synergistic and caused tumor growth suppression and in some cases complete remission^32^.

To select the most promising combination mechanisms for further detailed evaluation, we prioritized those that we considered truly synergistic, meaning where synergy scores were inversely correlating with cell viability. We calculated killrates for predicting the cell viability decreasing effect of the drugs (killrate = 1 - normalized survival). Some DDR inhibitors combined with compounds acting beyond DDR, were identified as strongly synergistic combinations in specific indications supported by an emphasized cell viability decreasing effect.

**Fig. 4:**
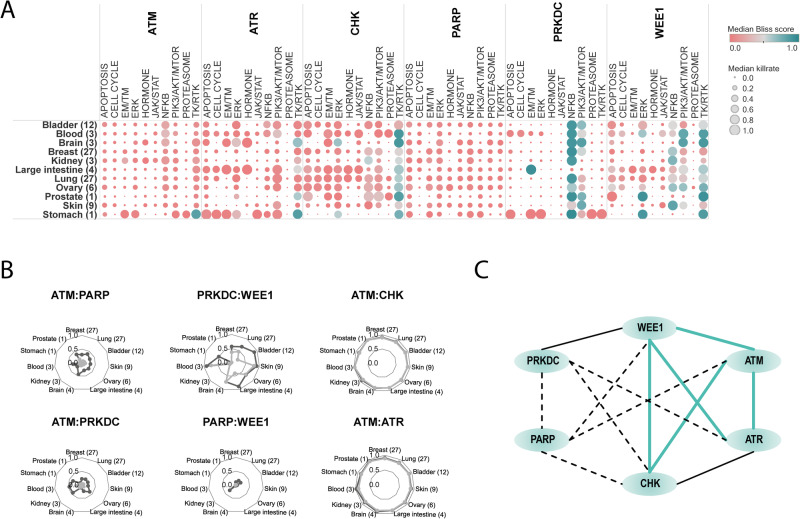
Systematic approach for in silico synergy profiling of drugs categorized into different mechanism of action (MoA) groups on cell lines representing various tumor types. **A** Heatmap for DDRi:non-DDRi combination synergies. Mean of synergy and mean of killrate were calculated for each MoA group for all cell lines in the certain indication group. Combinations that had synergy >0,5 were highlighted with turquoise color. The size of the circles represents the combination’s effect on cell viability. Bracketed numbers after the name of the indication at the left represent the number of cell lines in that group. The plot shows that CHKi combined with ERKi were strongly synergistic in brain tumor cell lines. PRKDCi represented a strong synergistic effect with EM/TM inhibitors. PRKDCi:NFKBi combinations were highly synergistic and at least cytostatic in many of the indications except for kidney, skin, and colon cancer cell lines. NFKBi performed well in combination with the WEE1 inhibitor only in kidney and melanoma cell lines. Protein synthesis inhibiting drugs (PIK3/AKT/MTORi) combined with WEE1i seems to be strongly synergistic in brain cancer cell lines only. **B** Indication-specific synergistic patterns of DDRi:DDRi combinations. Charts are representing some examples of non-synergistic (ATMi:PARPi, ATMi:PRKDCi, WEE1i:PARPi), moderately synergistic (WEE1i:PRKDCi), and strongly synergistic combinations (ATMi:CHKi, ATMi:ATRi). The latter combinations had a strong cell viability decreasing effect too in all indications, which suggests that ATM inhibitors in combination with ATR and CHK targeting compounds have a cytotoxic effect. Mean of synergy and killrate values were calculated for each MoA group for all cell lines categorized into indication groups. **C** Intra-DDR module combination synergy map reveals frequent intra-DDR module synergy except for PARPi and PRKDCi (except PRKDCi:WEE1i) combinations. Turquoise lines: strongly synergistic relationships between compounds where the cell-killing effect of the combination supported the synergistic phenomenon. Black continuous lines: moderately synergistic combinations, where an intermediate cell viability decrease was observed. Dashed lines: weak or non-synergistic combinations. CSNK Casein kinase, DDR DNA damage repair, EM/TM epigenetic/transcriptomic modulation, ERK extracellular signal-regulated kinase, JAK/STAT Janus kinase/signal transducers and activators of transcription, NFKB NF-kappa B, PIK3/AKT/MTOR phosphatidylinositol 3’ -kinase(PI3K)-AKT-mTOR, TK/RTK tyrosine kinase/receptor tyrosine kinase.

Interestingly, from the DDRi:non-DDRi combinations, PRKDCi:NFKBi inhibition performed with a strong synergistic effect in most of the cell lines and indications (Figs. 3, 4A), and therefore was prioritized for biomarker hypothesis generation analysis (Supplementary Discussion). Among the better performing DDRi:DDRi combinations, ATM, ATR, CHK inhibitors were observed to be highly synergistic with almost all the other DDR targeting compounds, while interestingly PARP inhibitors were identified as weakly synergistic in combination with other DDR agents (Fig. 4B, C). However, combination of PARPi and ATMi showed relatively strong killrate changes (Supplementary Fig. 7), indicating potential cell killing effect despite the lack of synergy. This observation, together with the clinical relevance of this combination, served as a basis for selecting it for further detailed evaluation.

### Drug combinations with protein synthesis inhibitors were most accurately predicted with DDR inhibitors

**Fig. 5:**
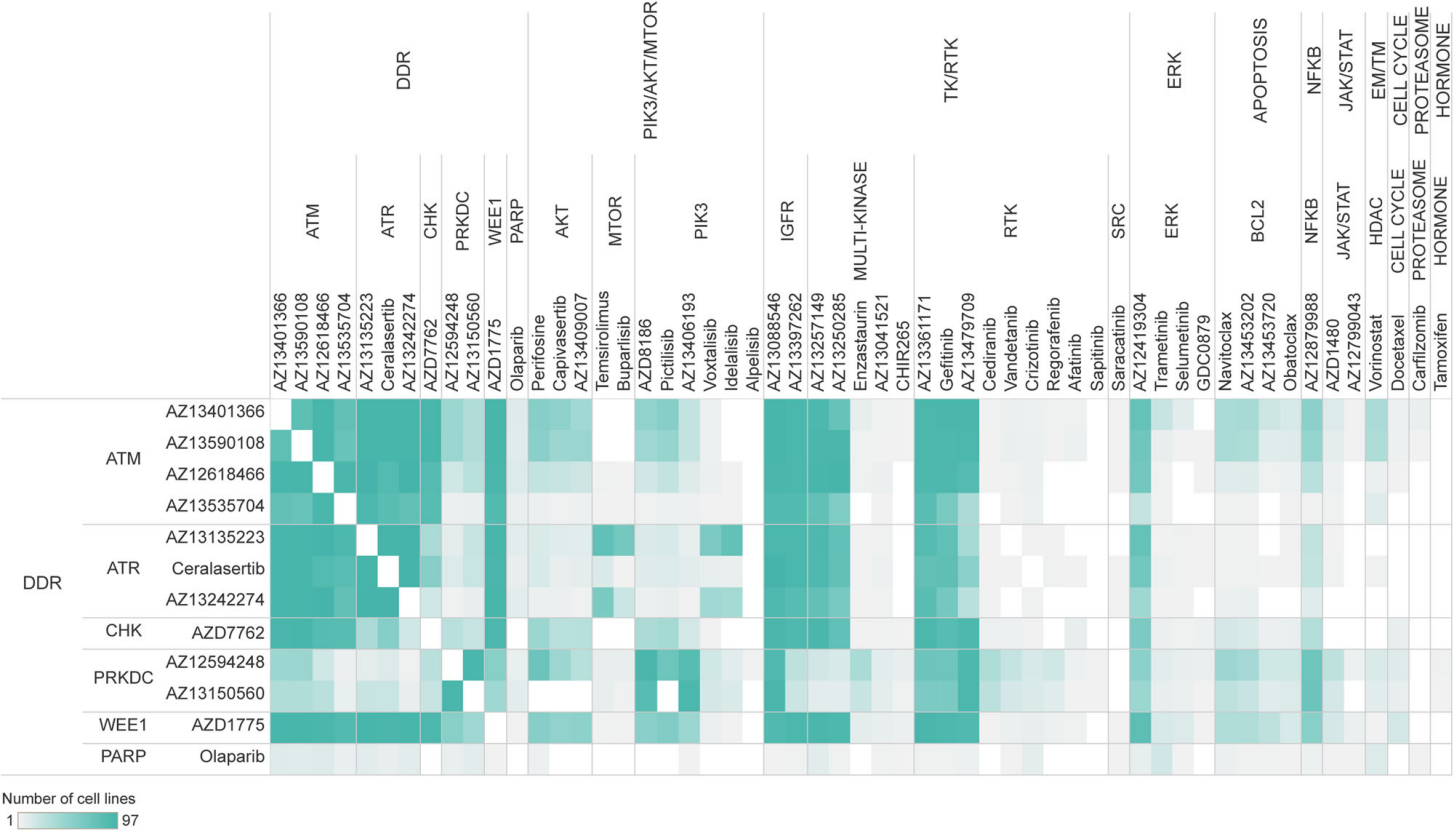
In vitro combination synergy measurements used for benchmarking analysis of in silico synergy predictions. The heatmap represents the number of cell lines in our in silico screen searching for potentially synergistic drug combinations, which overlap between the DREAM challenge and our dataset for each exact combination. The drugs were categorized into MoA groups based on the molecular targets of the compounds with the lowest binding affinity value. We had the best coverage of in vitro experimental data points in the case of DDRi:DDRi and DDRi:TK/RTKi combinations, also we managed to gather a good amount of data points regarding some DDRi:PIK3/AKT/MTORi combination-cell line pairs. For the rest of the combinations, coverage of in vitro measurements was poor.

In vitro experimental synergy values for our benchmarking analyses were collected from the DREAM challenge published dataset^17^. Out of the predicted 66,348 combination-cell line pairs, in vitro combination synergy values were available for a total of 977 combination-cell line pairs. The coverage of the in vitro cell response data differs between compound MoA categories, where availability of benchmarking data is generally superior for DDRi:DDRi over DDRi:non-DDRi combinations (Fig. 5).

**Fig. 6:**
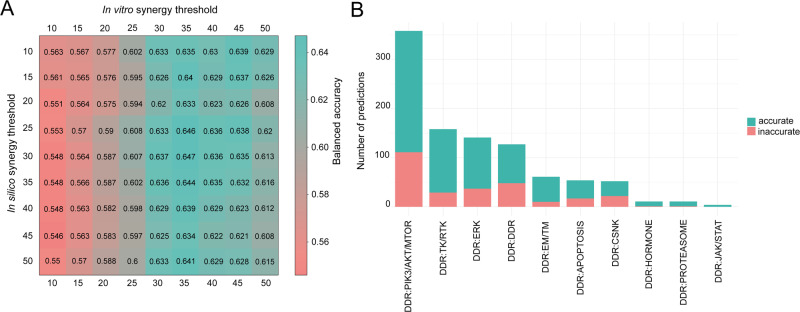
Benchmarking in silico combination predictions to in vitro synergy measurements. **A** In silico balanced accuracy across various synergy thresholds. Matrix represents the threshold value-dependent consensus (expressed in Balanced Accuracy, BA= (TPR + TNR) /2) between in vitro and in silico synergy. In the DREAM challenge, a threshold of 20 for both in vitro and in silico synergy was used to distinguish synergistic and non-synergistic combinations (Methods). However, the DREAM in vitro results were scaled between 0 and 100, while our in silico predictions are between 0 and 1, therefore the in silico threshold of 0.2 would correspond to it in this case. If we would use this cutoff value for the same endeavor as a gold standard, the balanced accuracy would be just under 0.6 (BA = 0.575, sensitivity=0.37, specificity=0.78). When we set the in vitro threshold for synergy to 30 and 20 for in silico synergy, our predictive power in balanced accuracy significantly improved (BA = 0.62, sensitivity=0.46, specificity=0.78). By increasing the in vitro threshold, the number of synergistic data points decreased, and the model was able to correctly detect a bigger proportion of synergistic combinations. This shows that in silico predictions are more accurate when stronger synergy is observed in vitro. **B** Proportion of accurate and inaccurate simulated measurements in different combination MoA groups. Due to the low in vitro data coverage, in many cases, benchmarking was not possible. Both in vitro and in silico synergy thresholds were set at 20, e.g., if both values were bigger or equal to 20, we considered that combination-cell line pair to be accurately synergistic and vice versa. Proportion of accurate and inaccurate simulations was calculated compared to the 977 overall data points. MoA groups are sorted based on the descending proportion of accurate simulations. CSNK Casein kinase, DDR DNA damage repair, EM/TM epigenetic/transcriptomic modulation, ERK extracellular signal-regulated kinase, JAK/STAT Janus kinase/signal transducers and activators of transcription, NFKB NF-kappa B, PIK3/AKT/MTOR phosphatidylinositol 3’ -kinase(PI3K)-AKT-mTOR, TK/RTK tyrosine kinase/receptor tyrosine kinase.

To assess the synergy prediction performance of our in silico model, we applied balanced accuracy (BA) as a metric (Methods). For BA calculations we used a synergy threshold of 20 for the in silico results (that would correspond to 0.2 on the original 0 to 1 scale) and 30 for the in vitro values from DREAM. For the overall predictivity, we calculated 0.62 for BAC and 0.7 for the AUC from the ROC analysis (Fig. 6A, Supplementary Fig. 8). We observed that drug pairs that were most accurately predicted in combination with DDR inhibitors were mainly protein synthesis inhibitors targeting the PI3K-AKT-MTOR axis, ERK, and other DDR-targeting compounds (Fig. 6B).

### Validation of the Simulated Cell on a machine learning benchmark framework

To assess the predictive performance of the Simulated Cell (model version 4 used in this study), we used synergy scores from the DREAM challenge as a benchmark. However, it was essential to establish a baseline performance metric to gauge the incremental value of our findings. Employing the introduced BA metrics outlined in the DREAM publication allowed us to determine the Simulated Cell’s performance in the context of synergy prediction. Nonetheless, while the DREAM challenge focused on directly predicting synergy scores using machine learning methods, our Simulated Cell models elucidate the molecular mechanisms underlying dose-dependent monotherapy responses and combination synergy scores. The methodology facilitated by the CellBox model^23^ naturally led us to adhere to the benchmark principles outlined in their work.

For evaluating the performance of synergy prediction, we employed three of the standard machine learning (ML) methods as baselines: (i) a fully-connected neural network model (NN)^33^; (ii) a regularized linear regression model (LR)^34^; and (iii) a gradient-boosted tree model (LightGBM)^35^. Consistent with the approach adopted in the DREAM challenge, we utilized the same approach for calculating BA using the above mentioned 977 overlapping in silico-in vitro combination-cell line pair dataset. Here, we also employed the precursory in silico synergy threshold of 20 for the ML methods and 30 for the in vitro values from DREAM. The overall predictive performance, as represented by the BA score, yielded values of 0.62, 0.52, 0.40, and 0.49 for the Simulated Cell, NN, LR, and LightGBM models, respectively (Supplementary Methods 4, Supplementary Fig. 12).

Although validating the synergy predictivity performance was our main goal, we also wanted to know whether the benchmark ML models have any advantage compared to the monotherapy IC50 prediction performance of the Simulated Cell. Therefore, we also estimated IC50 values based on the raw killrate output of all computational methods, and compared the correlation of the in silico values with the ground truth IC50s provided in the DREAM dataset. This resulted in a better performance of the Simulated Cell model in cell exclusive (CEX) and all exclusive test (AEX) set splits. In case of CEX split a mean IC50 correlation of 0.145, –0.011, 0.021, 0.028 for Simulated Cell, NN, LR and LightGBM methods, respectively. When the test set was established in AEX setup the yielded correlation values in the same order were 0.17, 0.026, 0.046, 0.041. In drug exclusive (DEX) split, LR had the ultimate advantage compared to the other models. These results showed us that incorporating biological prior knowledge into a computational model, like the manual calibration of the topology parameters can aid the generalization properties of predictive algorithms on unseen tasks (Supplementary Fig. 12). Thus, we hypothesize that this additional prior had a beneficial effect on the Simulated Cell’s performance for the combination prediction task.

### Involving more in vitro data enhances the predictivity of the Simulated Cell model

It is equally important to understand where the Simulated Cell model could provide reliable predictions and to identify the governing features of accuracy. In the DREAM challenge, some of the models favored specific subclasses of indications or combination groups. The results revealed that well-predicted classes represented combinations targeting DDR, together with receptor tyrosine kinase pathway and apoptosis inhibitors. We also observed in our analysis that combinations of these mechanisms mainly resulted in accurate predictions.

The Simulated Cell (model version 4 used in this study) was calibrated to monotherapy IC50 values for predicting synergy between given compounds and not on combination synergy values (like used in the DREAM Challenge), therefore it is important to evaluate what features drive an accurate synergy prediction. Besides the monotherapy accuracy, cell line coverage and the number of shared targets seemed to be the most important (Supplementary Methods 3, Supplementary Figs. 9–10), when we evaluated the 977 data points where both in vitro and in silico synergy values were available. In contrast, when we evaluated the main governing features behind a combination (predicted to be synergistic regardless of this being true in vitro), the results revealed the compound-target binding affinities and the monotherapy prediction accuracy for the DDRi compounds as the most relevant features based on all our in silico combinations (66,348 data points) (Supplementary Methods 3, Supplementary Fig. 11). These results demonstrate that involving more in vitro compound data and increasing the number of elements included in the protein interaction network has the potential to improve both monotherapy and synergy predictivity improvements.

Since our in silico signaling network includes several pathway cross-talks, our combination synergy prediction may perform well, even in cases where the monotherapy performance is not accurate. This assumption is supported by plenty of evidence on how drug combination synergies are affected by the hindered biological cross-talks of pathways downstream from the primary drug targets^24,36,37^.

### Molecular alterations that significantly shift synergy are involved in DDR pathways

Many drugs, either as a single agent or as combination therapy, fail in the clinic due to lack of efficacy. Therefore, the identification of biomarkers, predictive of patient response, became an essential part of the drug discovery process, drastically increasing clinical success rates^38^. We selected two drug combinations, PARPi:ATMi (Olaparib or AZD0156:AZ13535704) and PRKDCi:NFKBi (AZ13150560:AZ12879988) (Supplementary Discussion) as examples to generate a biomarker hypothesis through a signaling-level understanding of how combination benefit emerges behind the observed synergy and viability score changes.

PARP inhibitors are showing efficacy in the clinic with different biomarker patterns both in monotherapy and in combination. In our dataset, PARP inhibitors were represented with a homogenously poor synergistic effect in combination with other DDR and non-DDR targeting inhibitors. We selected this mechanism as an example to find molecular alterations that tend to shift synergy in a positive manner and have a more prominent cytotoxic effect on cell lines. Additionally, there is preclinical data underlining the potential of the PARP inhibitor Olaparib in combination with one of the ATM inhibitors included in our analysis^39–41^. Thus, we selected this combination to perform a detailed combination-specific biomarker screen.

The Simulated Cell model gives us the ability and flexibility to perturb the activity and concentration parameters of any node included in the signaling network. We simulate the effect of protein over- or under-expression on a given signaling pathway by multiplying or decreasing the concentration of a given node. By knocking it out or making a protein constantly overactivated, we simulate the consequence of hypomorph loss-of-function or hypermorph gain-of-function mutations. These modifications enable us to mimic given extrinsic modifications in a pathway and follow how the signal propagates from one protein to another before and after introducing a particular perturbation. This approach helps identify the biological mechanism behind the observed cell fate after drug exposure in different doses (Methods). After a systematic prescreen for protein alterations causing a combination-specific effect (Supplementary Methods 2), we selected 41 alterations consisting of 33 proteins or protein complexes for the PARPi:ATMi combination to analyze their effect on synergy and cell viability. We further analyzed only those combination-cell line pairs where the dose of the individual combination members at the maximum synergy score was lower compared to the IC50 value of the respective monotherapies, thus exhibiting the potential to decrease drug toxicity and increase drug efficacy. Furthermore, we excluded biomarker-combination-cell line triplets where a significant cell survival decrease was observed but the synergy shifted to a non-synergistic state, as these cases are suspected model artifacts.

**Fig. 7:**
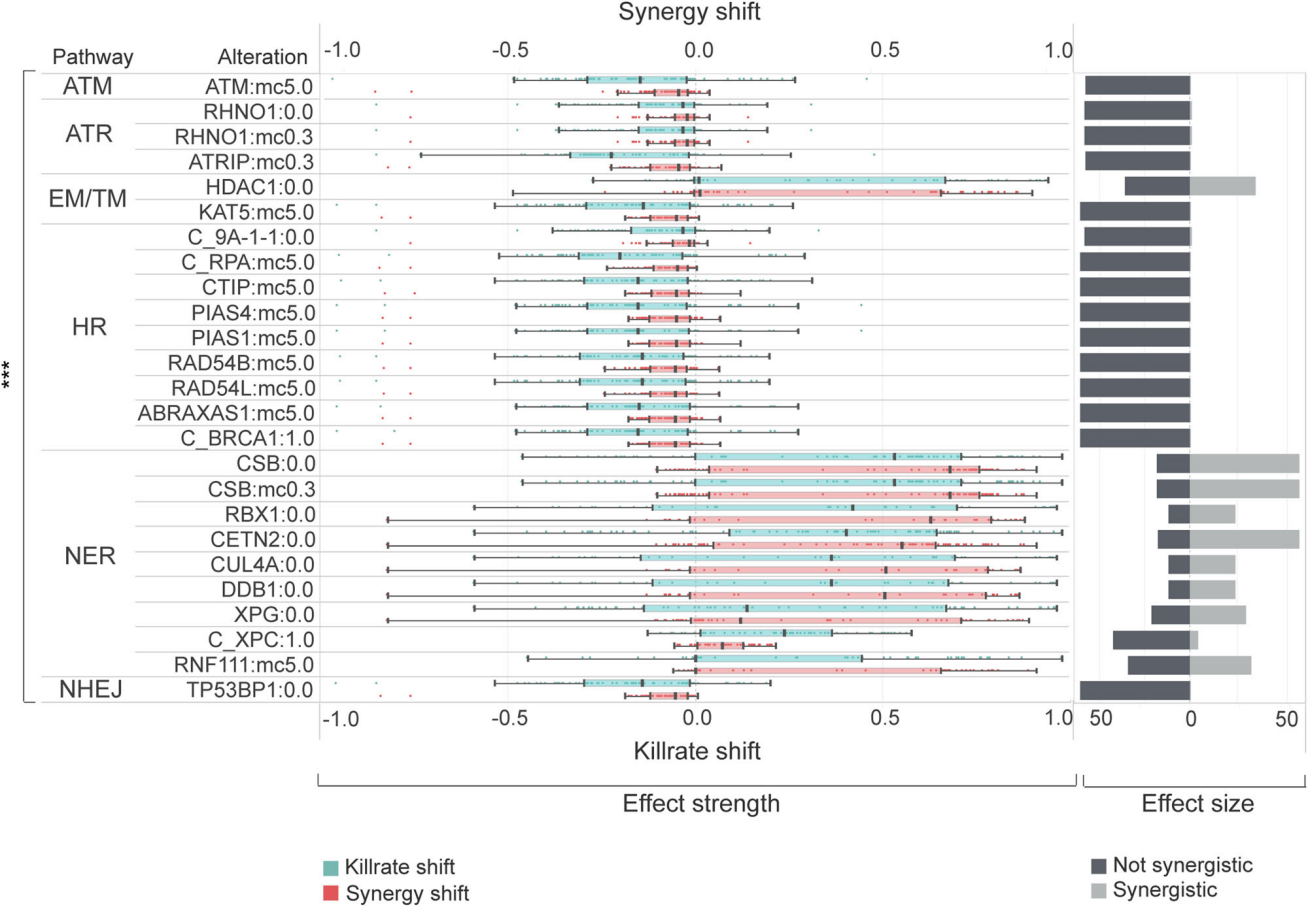
Effect strength and effect size of combination-specific biomarkers for ATMi:PARPi combination. This figure represents the statistically significant (*p* < 0.001, Wilcoxon rank sum test) shift of killrate and synergy influencing biomarkers in cell lines. Biomarker perturbation is an alteration that caps the maximum and or minimum value of the protein representing a node’s activity and concentration parameter. Combination-specific biomarkers can shift the synergy of a drug combination but failed to generate IC50 shift in terms of the monotherapy response of the corresponding drugs. Alterations for biomarker screening are described in detail in Supplementary Methods 2. Horizontal bars demonstrate the sample size for each biomarker, while the distribution of effect strength components (killrate shift and synergy shift) are represented in boxplots. The loss of NER genes has a favorable influence both on synergy and killrate, while the gaining of HR, ATM, ATR, and NHEJ members have a negative power on effect strength components. Legend: ***statistically significant *p* ≤ 0.001, 0.0 loss-of-function, 1.0 gain-of-function, mc5.0 overexpression, mc0.3 underexpression, C_ protein complex. ATM ataxia-telangiectasia mutated kinase pathway, ATR ATM and rad3-related pathway, EM/TM Epigenetic and transcriptomic regulation, HR homologous recombination, NER nucleotide excision repair, NHEJ non-homologous end joining.

**Fig. 8:**
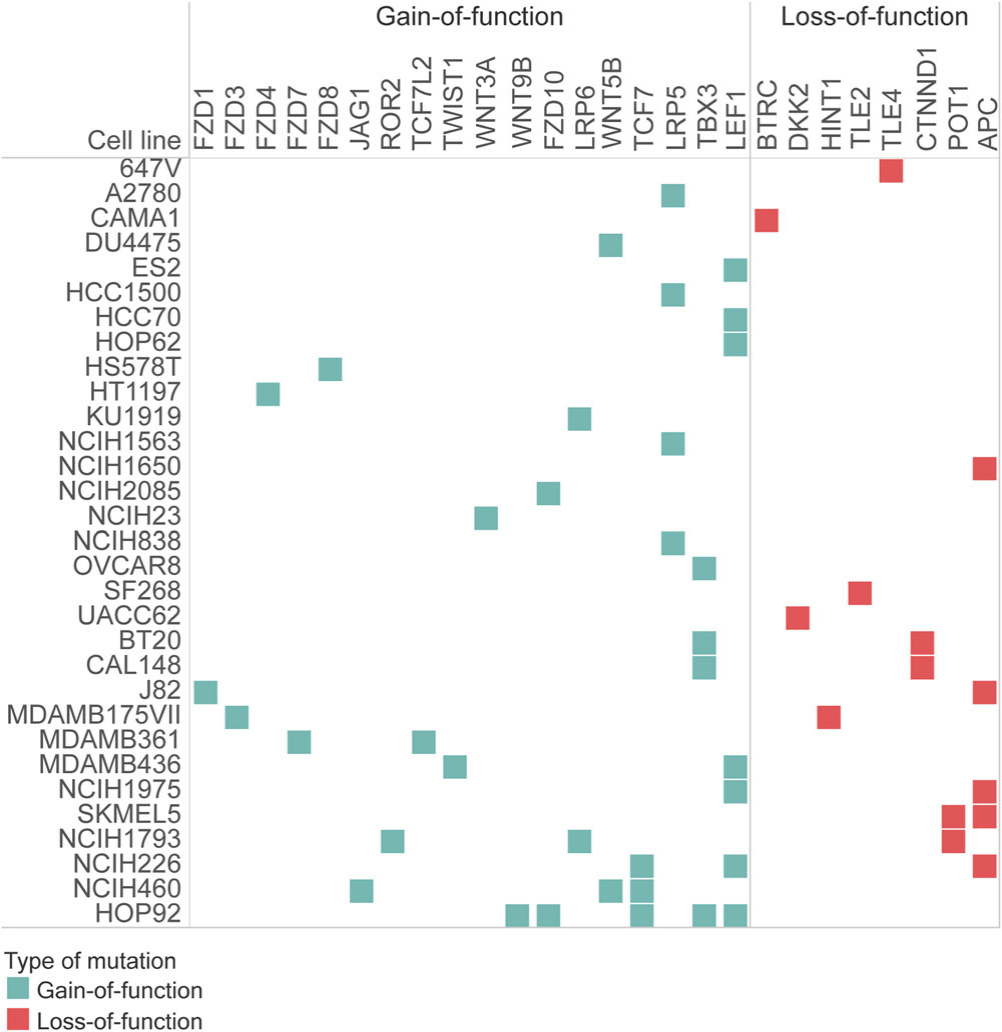
Cell lines, where HR pathway perturbation resulted in synergy and viability decrease had mutations entangled to the WNT pathway. Mutations of cell lines that decreased their viability after artificial perturbations mimicking overexpression and gain-of-function mutations of HR pathway members had a common, intrinsic WNT pathway entanglement. These mutant WNT pathway genes caused the overactivation of the WNT pathway, which is known to be a potential resistance mechanism to PARP inhibition. Gain-of-function mutations appeared in proteins important in activating the WNT pathway, while loss-of-function mutations tended to be appearing in negative regulators of the WNT pathway.

We observed that all the alterations resulting in a significant synergy shift are members of different DNA-damage repair pathways. Interestingly, alterations for decreased synergy (potential resistance) were enriched in given DDR pathways, such as homologous recombination (HR) (Fig. 7). In cell lines where HR pathway members were enriched regarding synergy decrease, more than 37% of them carried intrinsic damaging mutations in members of the WNT pathway, causing the pathway’s overactivation (Fig. 8). Many of the most promising alterations increasing synergy were members of nucleotide excision repair (NER), such as CSB, RBX, CETN2, CUL4A, DDB1, and XPG loss-of-function alterations. Inactivation of the epigenetic eraser HDAC1 also led to overactivation, a similar effect as NER pathway perturbation (Fig. 7).

**Fig. 9:**
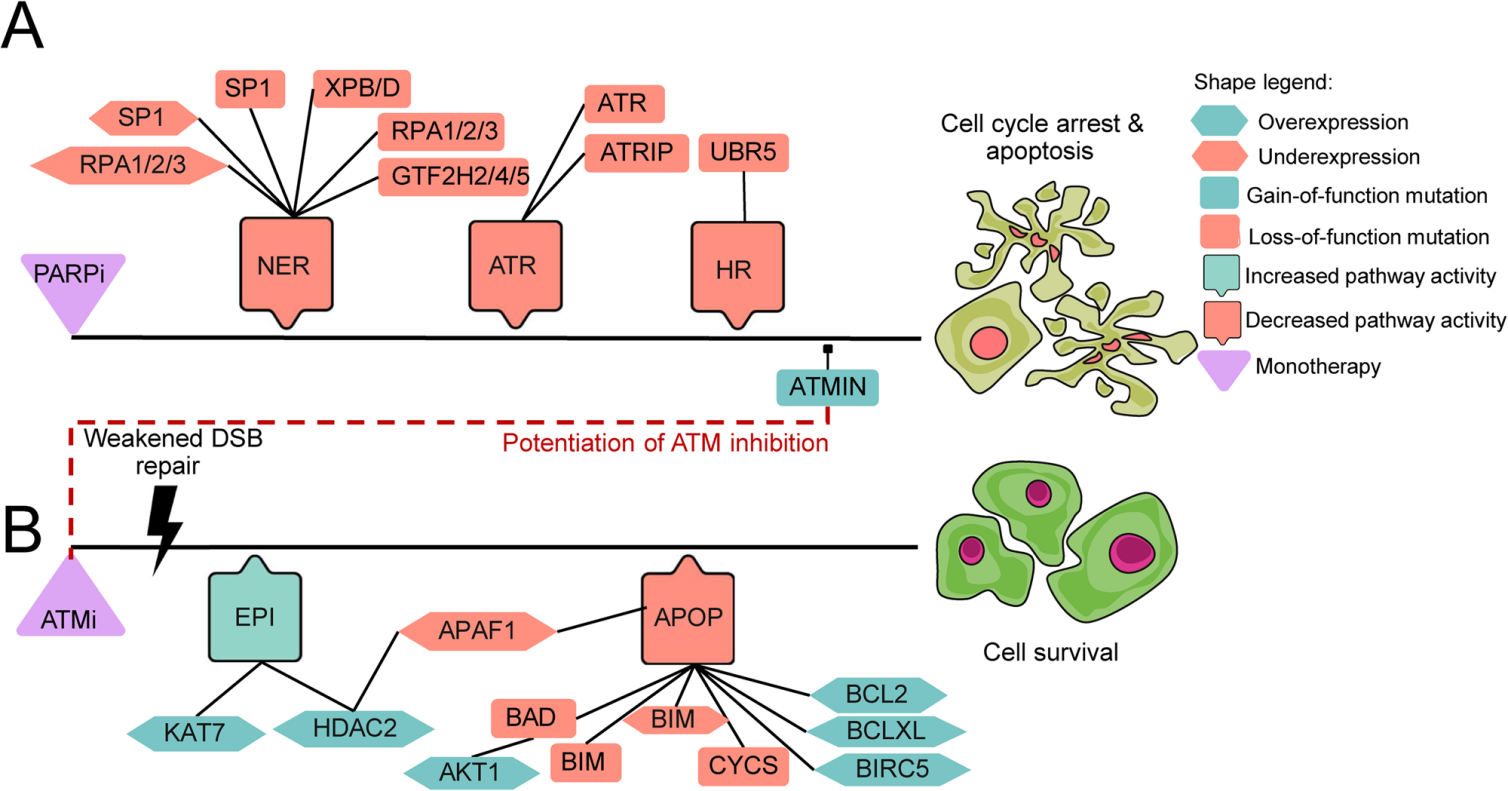
Signaling topology of detected alterations for ATMi and Olaparib can be followed based on the signal propagation in the Simulated Cell network. This visualization represents how each of our predicted monotherapy biomarkers could influence drug responses. Black line represents a theoretical upstream-downstream topology of detected biomarkers based on our signaling network. **A** PARP inhibitor-specific sensitizing biomarkers responsible for the downregulation of NER, ATR, and HR pathways which results in triggering cell cycle arrest and apoptosis. **B** ATM inhibitor-specific resistance biomarkers are upregulating some members of epigenetic regulation (EPI) which results in the effect of suppressed apoptosis (APOP). Further apoptosis downregulating biomarkers were also observed. Red dashed line represents the point in signaling where the benefit of combining the two chosen drugs could emerge. Since ATMIN gain-of-function surfaced as a sensitizing biomarker of PARP inhibition, this could explain how the combination can overcome the resistance that emerged upon ATM inhibition. The connection between the biomarkers is indirect and shows signal propagation from the drug target to the outcome only.

Besides the combination-specific biomarkers, we also analyzed the viability-changing monotherapy-specific biomarkers of the two compounds (Fig. 9, Supplementary Methods 1). In 6 cell lines, 20 molecular alterations were detected as resistance markers for ATMi specifically, while no sensitivity markers were observed. For PARPi, 15 protein alterations were observed as sensitivity markers in 6 cell lines (Fig. 9, Supplementary Data 3).

## Discussion

Our goal was to demonstrate the power of a signaling network-based simulation framework for predicting combination synergy and respective biomarker candidates. Our screen focused on the possible interplay between DDR-targeting drugs with other DDR and non-DDR compounds. We generated combination synergy scores for 684 drug combinations in 97 cell lines by simulating a calibrated signaling network, and without training on the combination data itself. We used the DREAM Challenge experimental dataset to benchmark the accuracy of our drug synergy prediction^17^. The benchmarking analysis demonstrated that the in silico simulation-based results are comparable to the top performers in the challenge (BA = 0.62, AUC = 0.7), even though the Simulated Cell (model version 4 used in this study) was not trained on any combination dataset before simulations.

Unfortunately, the benchmarking exercise could only be done on the in silico—in vitro overlapping dataset of 977 drug combination-cell line pairs, which limits the strength of the comparison. If we break down the non-DDR monotherapy results into MoA classes, among the best performers were drugs targeting tyrosine kinase signaling, the hormone receptor-mediated pathway, and apoptosis, while the worst performers were inhibiting extracellular signal-regulated kinase (ERK) signaling or targets like CSNK (Fig. 2D), indicating variability in predictivity among the different MoA classes. These results suggest that the model’s classification power is generally better regarding DDR inhibitors compared to non-DDR targeting drugs. This observation is in line with the aim of this study to establish accurate modeling of DNA Damage Repair to enable combination synergy and response biomarker predictions. In summary, we demonstrate the potential of the Simulated Cell technology to predict novel combination synergies on a large scale, which combined with the mechanistic explanation behind the observed synergy gives a solid ground for selecting combination treatments for experimental validation.

We also established a baseline ML benchmark framework, comparing the combination synergy and monotherapy response predictive performance of our model to three additional standard ML models. The Simulated Cell (model version 4 used in this study) had an advantage compared to those based on the calculated BA metrics (BA = 0.52, 0.40, and 0.49 for the NN, LR, and LightGBM models, respectively). To gain deeper understanding of the variations in performance levels, a benchmark analysis was also executed on the monotherapy responses. In order to estimate the IC50 values for each cell line-drug sample, sigmoidal curve was fitted, and a Pearson correlation was calculated to assess the performance relative to the published IC50 values of the DREAM challenge. When benchmarking the IC50 predictions of these models, we noticed worse generalization capability of the benchmark models compared to the Simulated Cell’s in CEX and AEX test splits according to the mean IC50 Pearson correlations. Kim et al. states that even if the data-driven solutions can aid computational modeling, but manual fitting procedures can produce additional values by applying experimental intuition and prior knowledge^42^. As node and edge parameters of the Simulated Cell’s signaling network – as driving factors of signal propagation – are also manually calibrated, we hypothesize that the Simulated Cell has the advantage of better prognosis on downstream tasks like synergy prediction and computational dose-response curve estimation.

The benefit of PARPi monotherapy treatment for HR-deficient tumors is already realized in the clinic and approved in high unmet-need populations such as pancreatic or ovarian cancer^43–45^. However, there is a relatively high chance of drug resistance both intrinsically and acquired^46^. Understanding the mechanisms behind the emergence of resistance prompts the discovery of novel, more precise biomarkers for patient stratification, and could also lead to innovative combination strategies. ATM is not just a patient stratification marker in prostate cancer for PARPi therapy^47^, but also a potential target for a combination approach^31^. However, identifying the precise patient population to target with this DDRi:DDRi combination could be essential to limit potential toxicity^41^.

As combination synergy itself is indicative but not enough to reach combination benefit in the clinic^14,48^, we aimed to generate further insight into the combinations by observing the cell-killing effect and hypothesizing biomarkers of increased sensitivity or potential resistance to the drug combination. These results remain to be experimentally validated but the potential of understanding why synergy emerges and how we can use this information for patient selection is demonstrated in two examples.

We hypothesized that combination synergy would appear when PARPi-specific perturbations overcome resistance mechanisms for ATMi, from which ATMIN gain-of-function mutation could have a crucial role. The loss-of-function of XPB or XPD helicases along with GTF2H2/4/5 could sensitize certain cells to such inhibitors. A loss-of-function mutation of RPA proteins will prolong the initiation of the damage signal by missing the activation of repair inducer ATR at single strand-break sites. Gain-of-function mutations of ATMIN appeared in our prediction because of its ATM inhibiting feature, therefore combining PARP inhibitors with drugs targeting ATM could emerge as a potential combination strategy. ATM inhibition increases the number of double-strand breaks that will grow in a tumor population since the underlying damage response is not initiated, activating apoptotic signaling after a certain limit of errors has been reached. Therefore, underexpression of apoptosis inducers or overexpression of anti-apoptotic proteins could be a potential biomarker. We also predicted that AKT1 upregulation can override the cell viability, decreasing the effect of DNA damage upon ATM inhibition^48^. Epigenetic apoptosis regulation by APAF1 via HDAC2 or KAT7 could also be a potential biomarker, supported by our predictions as well as literature evidence^49,50^.

In most of the cell lines treated with the Olaparib:ATMi combination, independent from their tissue of origin, the introduction of deficient NER proteins increased synergy. CSB is considered to be a master regulator of transcription-coupled nucleotide excision repair (TC-NER) as it plays a key role in the recruitment of several proteins to the TC-NER complex^51^. XPG is another indispensable core protein of the NER machinery, whose function is to cleave the DNA strand at the 3′ side of the DNA damage^52,53^ and is essential for the 5′ incision by the ERCC1/XPF endonuclease. CUL4A, DDB1, and RBX1 are core components of the cullin-RING-based E3 ubiquitin-protein ligase (CRLs)^54,55^ complexes which physiologically can promote ubiquitination labeling and proteasomal degradation of target proteins involved in cell cycle progression, transcription, and TC-NER^56^. CETN2 is a crucial factor in global genome nucleotide excision repair (GG-NER) by acting as an element of the XPC complex^51,57^. This protein assembly is proposed to be one of the first to bind to DNA damage sites and together with other core recognition factors, XPA, RPA, and the TFIIH complex, is part of the pre-incision complex recognizing single-stranded DNA lesions^58^. The lack of these crucial components of TC- and GG-NER machinery might be a suitable predisposal factor for impairing single-strand break repair. Since PARP and ATM targeting generate a synergizing mechanism on DSB repair downregulation, increasing the cell’s mutational load will begin apoptosis^59^.

Beyond identifying NER-related biomarkers shifting cellular response to a more synergistic manner, we also identified the overexpression of HR pathway members (BRCA1 complex, CTIP, RAD54B/L, PIAS1/4, ABRAXAS) leading to decreased efficacy of the combination in cell lines where moderate synergy was detectable. We hypothesize that increased HR activity allows cells to balance between constantly increasing mutational load and decreasing genome integrity, resulting in an increased fitness against the dual intervention of PARPi and ATMi.

Interestingly, the intrinsic mutational profile of these resistant cell lines shared a common feature, namely functionally damaging alterations on the upstream part of WNT signaling. FZD3/4/10 gain-of-function mutations might cause the WNT ligand receptor, Frizzled, to be constantly activated leading to the inactivation of the destruction complex and subsequent translocation of β-catenin into the nucleus followed by the positive regulation of WNT-dependent transcription^60^. While FZD3 overexpression was associated with malignancies like lung cancer, FZD4 and FZD10 upregulation was frequently observed in pancreatic and colon cancer, respectively^61–63^. Interestingly, these resistant cells represented targets (LEF1, TLE2/3) of the WNT pathway being also affected by damaging mutations. LEF1 overactivation serves as a good breeding soil for the upregulation of factors having a crucial role in cell proliferation regulation, like c-MYC and CCND1^64^.

WNT upregulation is a known mechanism in drug resistance, especially in the case of PARP inhibitors^65^. Restored HR pathway activity by for example having function-correcting secondary mutations in BRCA1, managing for the protein to form complexes together with such recombinational well-studied cofactors like the MRN assembly and its further cofactor proteins are well-known resistance biomarkers upon Olaparib treatment, but not reported in combination with ATM inhibition before^46^.

In order to translate these in silico predicted biomarkers to the patient level, first, we estimated the number of patients who could benefit from the Olaparib:ATMi combination therapy. According to our appraisal, more than 120,000 patients with various tumor types could benefit from the combination (Supplementary Data 5). We also analyzed the prevalence of the predicted biomarkers to identify segments of the patient population with a higher likelihood of response. Based on the genomic and transcriptomic data of 30 TCGA PanCancer studies, alterations of ATRIP, CUL4A, DDB1, RAD54B/L, and TP53BP1 were the most frequent, indicating their potential to be utilized as patient stratification biomarkers predicting the effectivity of the recommended Olaparib:ATMi combination (Supplementary Data 5).

In order to show that our approach could not just identify already known combinations, but also predict novel combinations and respective biomarkers, we selected the highly synergistic PRKDCi:NFKBi combination for further evaluation. Whilst there are several reports in the literature of PRKDC and NFKB inhibitor monotherapy activity, we did not find any information about whether these two compounds would potentiate each other’s effectivity or what are the downstream signaling effects of these drugs in combination. We predicted a dozen potential combination-specific molecular alterations that could shift the synergy and the cell viability beneficially or unfavorably (Supplementary Discussion, Supplementary Data 4).

In summary, we demonstrate the combination synergy predictivity of our signaling network model-based simulation framework, the Simulated Cell (model version 4 used in this study), benchmarking its performance compared to other prediction algorithms in a recent DREAM challenge^17^. Furthermore, we show that one of the main limitations of such algorithms could be overcome with the Simulated Cell model and offer easy-to-interpret mechanistic insights behind the emergence of synergy and ultimately increased cell-killing effect. Based on two examples, the combination of PARPi and ATMi, and the combination of PRKDCi and NFKBi, we were able to not just recapitulate known synergistic effects and underlying molecular features but also predicted novel combinations with a translatability potential based on the identified molecular drivers of synergy.

The Simulated Cell (model version 4 used in this study) also has its limitations as it requires relatively resource-intensive manual extension of the network, and manual calibration to near each monotherapy in silico result to the in vitro counterpart. This unfolds mainly in the phenomenon where protein-level biology can be recapitulated in silico better than cell survival verdicts. This problem could be solved by the development of a semi-automatic system to extend the network under human supervision and by training the model algorithmically utilizing machine learning. There are also further limitations around the translatability of the results. Limited information is available about the target profile of the drugs, which results in a relatively stronger influence of the primary targets while unknown further polypharmacological effects through potential off-targets are not captured. In silico avatars of cancer cell lines are bearing with the general translatability challenges of in vitro cancer models, however, the possibility to model the effect of extrinsic molecular alterations which are prevalent in patients is an advantage. Predicted individual transcriptional biomarkers are challenging to translate as these are more dynamic and dependent on environmental factors, therefore complementing the analysis with patient data-based expression analysis is needed, where the data is often limited compared to mutational prevalence.

Overall, we could demonstrate that this framework could be employed for de novo combination and biomarker predictions, enabling the selection of experiments to run on a more sophisticated starting hypothesis.

## Methods

### Concept of the Simulated Cell

The Simulated Cell (model version 4 used in this study) from Turbine Ltd. is a dynamic model of intracellular signaling that integrates cell line-specific information into a manually curated signaling network. This network comprises nodes representing unique proteins tagged with unique UniProt IDs^66^, along with a small number of logical nodes representing biological processes (e.g., DNA repair). Interactions between these nodes are directed, with inhibitory or activatory effects denoted by negative or positive edge weights, respectively. While base node characteristics being not cell line-specific parameters are partially defined by manual calibration, genomic/transcriptomic data informs and is able to modify these base parameters specific to a given cell line. Concentration and activity of nodes are treated as separate variables both at the outset and during simulation.

Perturbations propagate through the network via edges, with interaction strength described as a function of node activity and concentration at any given time, along with edge weight. Edge effects, combined with manually set base values, establish logical gates that determine downstream node activation by upstream regulators (Supplementary Methods 2). Specific compounds can be modeled by inhibiting or activating their molecular targets based on the magnitude of binding affinity constants and compound dosage. The Simulated Cell facilitates efficacy testing of compounds and augments results with insights into biological signal propagation from drug targets through downstream proteins to effector proteins determining cell fate (apoptosis or cell cycle) (Fig. 1).

### Structure of the signaling network

The Simulated Cell’s signaling network (model version 4 used in this study) was created manually by biologists based on trustworthy literature data, which is a common way for creating interactome datasets, such as in the Signor database^67^. Our network has several modules representing pathways (Fig. 2B). The network includes 56 modules covering the main cancer-driving pathways with a total of 1997 nodes and 5004 interactions, out of which 14 modules cover the DNA Damage Response related mechanisms (see Fig. 2B for more details). This connectivity map enables the modeling of module crosstalk and hierarchy in the Simulated Cell.

An ODE network has continuous-valued, continuous-time equations, whereas inside the Simulated Cell (model version 4 used in this study), the model consists of discrete-time, continuous-valued equations. This creates the possibility to model the effect of a protein representing node derived from the binary genomic variations along with the continuous expressional features. For each experimental setup, multiple simulations are initiated incorporating multiomic data from a specific cell line and various additional perturbations. These perturbations encompass a specified dosage of a compound as well as unique manually added perturbations such as gain-of-function mutations. These simulations exhibit intratumor heterogeneity due to introduced random noise artificially embedded into the activity and concentration parameters of each node. After a given number (usually 300) of time steps, 99.9% of the trajectories evolve into a stable attractor state^68,69^. Attractors are generated by matching specific node activation patterns with associated phenotypes. For all of these phenotypes, apoptosis and cell cycle score can be calculated based on effector output nodes’ activity that can be easily used as the indicator of the alive or dead status of a simulated cell. For example, the DNA fragmentation factor is irreversibly activated when the cell is already dedicated to initiating apoptosis in a specific cellular context and the activity of this protein can be used as the indicator of the activity of apoptosis. The correspondent nodes of apoptosis or cell cycle can reach values between 0 and 1 representing an inhibited (0) or an activated (1) state depending on the upstream signaling events. The Simulated Cell signaling is wired in a way that the cell can never be alive if apoptosis is active, even when its cell cycle is not disrupted. The software predicts a cell to be alive if its readout nodes representing the cell cycle reach at least 60% of maximum effect and apoptotic proteins are not activated at more than 20% of maximum effect. The readouts of the separate simulations with the same base characteristics beside random noise are summarized to predict the alive or dead status of a given cell perturbed by a given compound.

### Transcriptomics data processing

The Simulated Cell has cell lines from the Cancer Cell Line Encyclopedia (CCLE) in its off-the-shelf library. The transcriptomic input for these cell lines is differential gene expressions between CCLE^70^ cell lines and non-tumorous tissue data of Human Protein Atlas (HPA)^71^, a set of 200 samples derived from 32 different (healthy) human tissues. CCLE RNA-Seq data comes from the GDC Legacy Archive, while the HPA data comes from the EMBL-EBI ArrayExpress public repository under the accession ID of E-MTAB-2836^72–74^. Thus, the fold changes estimated the relative amount of the given mRNA in each cell line, compared to an average cell used as a baseline, and derived from the healthy expression profiles representing 32 different tissues. At the zeroth step during each simulation, the calculated foldchange value for each protein in each cell line is multiplied by the hand-calibrated base concentration. This way the relative abundance of each protein is represented in the model.

### Determination of genetic variants

In order to use both genomics and transcriptomics from the same source, the Simulated Cell uses genomics from CCLE, in particular from the DepMap (19Q3) mutation dataset^75^, converted to genome coordinates of GRCh38 by using CrossMap^76^. Next, its pipeline uses Ensembl Variant Effect Predictor^77^ (VEP) (we used the parameters listed in Supplementary Methods 3). The Simulated Cell’s input pipelines use this information to map functional consequences to each variant, first using ClinVar (version 2019–07)^78^, and failing that, predicting using metaSVM and metaLR ensemble scores^79^ from the commercial version of dbNSFP^80^ (v3.5). In the Simulated Cell, each node is manually annotated as behaving predominantly as a tumor suppressor or an oncogene. Pathogenic mutations hitting oncogenes are assumed to be gain-of-function mutations (unless they are clearly loss-of-function mutations like an early frameshift or stop gain) while pathogenic mutations hitting tumor suppressors are predicted to cause loss of function. The calculated effect of a specific mutation then modifies the achievable minimum or maximum value of the activity parameter of each affected node by either lowering the potential upper threshold to zero in case of loss-of-function mutation or elevating the lower threshold to one if it is a gain-of-function mutation.

### Compound target profile and cell response data

Bioactivity data for the compounds of interest were obtained from ChEMBL^81^ and additional filtering steps were conducted on the raw data. Firstly, we selected those data points that were derived from *Homo sapiens*. Secondly, we kept data points with confidence scores higher than 5 and consisting of binding assay type. This latter step keeps those data points that provide information about binding affinity. IC50 values above 10.000 nMol were considered ineffective and uncapped data (relation ‘>’) proved to be unreliable for our study, thus, both types were excluded. Additional drug target profiles that were not available in the ChEMBL database and were relevant for this analysis were acquired from AstraZeneca.

In vitro compound effects on cell lines have been also received from AstraZeneca. To achieve a greater coverage for the compounds we also downloaded IC50 values from various public drug databases (Supplementary Methods 1, Supplementary Data 1). The introduction of a drug onto an in silico cell line will result in a lower achievable maximum activity threshold as the concentration of a given drug increases.

### Summary of simulation types

The simulations on the Simulated Cell are running in a flexible, easily expandable platform. We summarize the different types of in silico experiments, in Table 2. For more details, please see Supplementary Methods 2.

**Table 2:** In silico simulation portfolio of the Simulated Cell

Experiment	Purpose	Input parameters	Output parameters
Native simulation	To measure the native phenotypic behavior of the cell lines without intervention	• Node parameters: base activity, base concentration • Edge parameters: direction, type, strength	• Node effects • Attractor scores • In silico verdict on cell fate
Monotherapy response screen	To measure the response of a cell line to a single drug intervention	• Native simulation input parameters • Molecular target profile of compounds	• In silico predicted IC50 values of cell lines/drug • Cell viability
Combination therapy screen	To measure the synergistic effect of two compounds in given dose points	• Monotherapy response input parameters • Dose grid	• In silico predicted synergy scores • Doses in synergistic points • Cell viability
Combination specific biomarker screen	To measure the possible effect of node parameter alterations on synergy	• Combination therapy screen input parameters • Altered node parameters: Under- and overexpression, Loss-and gain-of-function mutations.	• Shift of synergy scores by node parameter perturbations • Shift of cell viability by node parameter perturbations

By using the Simulated Cell, we are able to interrogate the in silico simulatable tumor cells to gather information about their native, untreated behavior and the dose-dependent mono- and combination therapy sensitivity while introducing additional single-node perturbation to the system.

### Manual calibration of the Simulated Cell

Native cell lines are expected to be in a proliferative alive state to mimic the in vitro proliferative behavior. To achieve this state, we adjust the base concentration and activity values of the individual nodes and the edge weight of the interactions. However, we never change the direction and the sign type of any edges. Throughout the manual calibration process, we scrutinize both the genomic and transcriptomic data layers, as well as monitor the activity and concentration variables during the simulation. In the cell line-specific genomic and transcriptomic datasets, we are looking for characteristics that can be related to its proliferative state. As an example, if a cell line harboring a dysfunctional retinoblastoma 1 tumor suppressor gene is deemed non-viable, we enhance the inhibition of the retinoblastoma 1 protein on the activity of the E2F1 transcriptional factor. This adjustment is made to amplify the impact of the loss-of-function mutation on cell cycle activity. Despite the genomic and transcriptomic features linked to the proliferative state of cell lines, and notwithstanding the prioritization of outgoing edges related to these nodes, if the cell is still marked with a “DEAD” verdict, we conduct dynamic simulation steps to pinpoint the precise juncture where the transmission of the proliferative signal is interrupted. This process can be repeated for all the cell lines with native dead verdicts. The edge weight changes are not cell line specific but are uniform in all the simulations.

Manual calibration, to fit the in vitro monotherapy response, is similar to native calibration. This process involves revisiting the baseline characteristics of the cells and scrutinizing the step-by-step signaling dynamics during dynamic simulation. We additionally examine the IC50 profiles of the drugs’ targets in our efforts to enhance the results of the simulations. From a biological standpoint, the in silico replication of the most sensitive cell line and most resistant cell lines’ drug response is the most important. For instance, in the case of Gefitinib, typically classified as an EGFR inhibitor, it exhibits an off-target profile where, despite possessing higher inhibitory constants, it also disrupts signaling from other receptor tyrosine kinases such as ERBB2/4 and FLT3. If one of the resistant cell lines had a gain of function mutation in one of these receptor tyrosine kinases (RTKs), the outbound edge weights of these RTKs could be increased so that in these cell lines, the inhibitions of these off-targets drive the cell killing effect. In case of extreme resistance, often non-targeted RTK or a protein member of a completely different pathway is mutated or has massive expression change. Similar to the native calibration, if the adjustment of direct edges from the nodes does not translate to a desired in silico readout, step-by-step evaluation during the simulation and identification of the signaling blockage is attempted. Given the extensive array of simulated cell lines and compounds, coupled with crosschecking the in vitro datasets, it is presumed that the various signaling pathways will exert appropriate relative effects on cell cycle progression, apoptosis, or any other phenotypes to which the model is calibrated. Due to increased sensitivity and the lower number of datapoints in the DREAM monotherapy dataset versus other public sources (Supplementary Fig. 3, Supplementary Data 1), we have been focusing the manual calibration on recapitulating the public monotherapy IC50s.

### Evaluation of Bliss independence-based combination synergy metrics

The Bliss independence model is a commonly applied statistical model to evaluate compound efficacy in combination through quantification of synergistic/antagonistic effects using the addition law of probability theory (Supplementary Methods 2)^31,82^. There are multiple reasons why using the Bliss independence model is preferred over other alternative synergy models like applicability on entire dose ranges and in case of non-standard or not available dose-response curves, straightforward probabilistic interpretation^83,84^. As our main interest was synergy, we modified the Bliss score in a way that we considered every negative Bliss score as zero (total lack of synergism) during the calculation of the aggregated statistics of Bliss scores (Supplementary Methods 2), therefore the probability of antagonism was not assessed in this study.

The benchmarking analysis of in silico and in vitro monotherapy and combination performances was evaluated by ROC. The predicted Bliss_max_IC50 (Supplementary Methods 2) scores were evaluated in comparison with the in vitro Bliss scores. To obtain binary classified in vitro measurements, we dichotomized the experimental scores into synergistic and non-synergistic classes using a synergy score threshold of 20. For visualizing and comparing ROC curves the pROC package^85^ was used in R.

The accuracy of AstraZeneca’s in vitro and our in silico combination screens were evaluated by a balanced accuracy metric. For the evaluation of combination results, first, an algorithmic method was chosen to discretize the continuous synergy scores. K-means clustering was applied which is a widely used discretization method in biological data analysis^86,87^.

The statistical significance of the difference between combination synergies aggregated by the compound mechanism group was established by pairwise Wilcoxon rank sum test with Benjamini-Hochberg correction. To test the statistical significance of combination-specific biomarker effects Wilcoxon rank sum test was applied. We concluded statistically significant those effect sizes that got *p*-value less than 0.001.

### Screening synergy prediction power of the in silico model

Synergy scores were binarized for comparison since only Loewe synergy scores were available to be compared to our Bliss independence scores. These two metrics tend to be different depending on the sigmoidicity of the dose-response curves^88^. Goldoni et al. identified the Hill coefficient as governor of this issue in case of high sigmoidicity, resulting in the overestimation of synergism by the Bliss independence model, while the Loewe additivity model overemphasizes antagonism^89^. Another difficulty in the comparison is the different calculations of the in silico and in vitro synergy values. We searched for the maximum value over the whole dose grid, while in the DREAM challenge, the sum of the synergy scores for the dose points is calculated. Based on this information, we applied balanced accuracy (BA) as a metric to evaluate the synergy predictions. For BA calculations, we used a synergy threshold of 20 for the in silico results (that would correspond to 0.2 on the original 0 to 1 scale) and 30 for the in vitro values from DREAM. Regarding the ROC analysis, the same in vitro threshold (30) was used.

### Machine learning benchmark pipeline

To deconstruct the inhibitory effects of drugs on their targets, we identified these targets from publicly available datasets. Leveraging their target-specific inhibitory constants, we computed their dose-dependent inhibition rates. Regarding the characterization of simulated cell lines, we utilized the CCLE transcriptomics dataset, concatenating it with drug target data to serve as inputs for the chosen standard machine learning methods. Thus, we propose that this benchmark setup enables a fair comparison of performance between machine learning methods and the approach of the Simulated Cell (Supplementary Methods 4).

Since the Simulated Cells model is calibrated primarily based on publicly available monotherapy responses and not on the DREAM dataset, we employed the DREAM dataset as a test set for evaluating Simulated Cell-derived model predictions. For the machine learning-based benchmark, we use monotherapy dose-response curve data for training the models, running ten random train-test splits. Three splitting strategies were applied for performance evaluation: along the dimensions of cell line, drug, and cell line-drug combination. To evaluate performance metrics for Simulated Cell model predictions, we calculate metrics for each test set created for linear ridge regression, a fully-connected neural network, and a gradient-boosted model (LightGBM). We consider that these models are broad enough to yield reliable performance evaluations. Regarding the monotherapy performance benchmark, we examine correlations between IC50 scores fitted on the Simulated Cell and machine learning predictions against DREAM in vitro IC50 values. Concerning combination therapy, we operate with balanced accuracy over in silico Bliss predictions and DREAM synergy scores. This metric evaluates both sensitivity and specificity of methods while considering the low number of synergistic cases. It is important to highlight, that for combination prediction all methods utilized the whole DREAM monotherapy dataset for training, and used the DREAM combination dataset only as test set.

### Supplementary information

Supplementary Material

Supplementary Data 1

Supplementary Data 2

Supplementary Data 3

Supplementary Data 4

Supplementary Data 5

## Data Availability

We made all relevant cell line, compound target, and in vitro cell response data, along with the in silico predicted monotherapy, combination, and biomarker datasets available in the Supplementary Material. The Simulated Cell’s input features, including the interactome as well, together with the simulation software are legally protected intellectual property of Turbine Ltd. In case of any further inquiry please contact the corresponding author.

## Code availability

The relevant codes used for data analysis are available in the GitHub repository: https://github.com/turbine-ai/az1-pub-simcell-combination-synergy.
