# Author Correction: Network-driven cancer cell avatars for combination discovery and biomarker identification for DNA damage response inhibitors

**DOI:** 10.1038/s41540-024-00416-7

**Published:** 2024-08-08

**Authors:** Orsolya Papp, Viktória Jordán, Szabolcs Hetey, Róbert Balázs, Valér Kaszás, Árpád Bartha, Nóra N. Ordasi, Sebestyén Kamp, Bálint Farkas, Jerome Mettetal, Jonathan R. Dry, Duncan Young, Ben Sidders, Krishna C. Bulusu, Daniel V. Veres

**Affiliations:** 1 Turbine Simulated Cell Technologies, Budapest, Hungary; 2 Oncology Bioscience, Research and Early Development, Oncology R&D, AstraZeneca, Waltham, MA USA; 3 Early Data Science, Oncology Data Science, Oncology R&D, AstraZeneca, Waltham, MA USA; 4 Search and Evaluation, Oncology R&D, AstraZeneca, Cambridge, UK; 5 Early Data Science, Oncology Data Science, Oncology R&D, AstraZeneca, Cambridge, UK

**Keywords:** Computational biology and bioinformatics, Computer modelling, Cancer

Correction to: *npj Systems Biology and Applications* 10.1038/s41540-024-00394-w, published online 21 June 2024

In the Acknowledgements section of this article, the team at AstraZeneca is referred to as the Early Computational Oncology team at AstraZeneca but should have been the Early Data Science team at AstraZeneca. Additionally, the name of the Co-Author Jay Mettetal should have been listed as Jerome Mettetal. Also the Supplementary File has been replaced. The original article has been corrected.

